# Diagnostic and treatment delay among Tuberculosis patients in Afar Region, Ethiopia: A cross-sectional study

**DOI:** 10.1186/1471-2458-12-369

**Published:** 2012-05-23

**Authors:** Mulugeta Belay, Gunnar Bjune, Gobena Ameni, Fekadu Abebe

**Affiliations:** 1 Aklilu Lemma Institute of Pathobiology, Addis Ababa University, Addis Ababa P.O.Box 1176, Ethiopia; 2 Section for International Health, Department of Community Medicine, Institute for Health and Society, University of Oslo P.O.Box 1130, Blindern 0318 Oslo, Norway

**Keywords:** Tuberculosis, Patients’ delay, Health system’s delay, Afar Region, Ethiopia

## Abstract

**Background:**

TB is a major public health problem globally and Ethiopia is 8^th^ among the 22 high burden countries. Early detection and effective treatment are pre-requisites for a successful TB control programme. In this regard, early health seeking action from patients’ side and prompt diagnosis as well as initiation of treatment from the health system’s side are essential steps. The aim of this study was to assess delay in the diagnosis and treatment of TB in a predominantly pastoralist area in Ethiopia.

**Methods:**

On a cross-sectional study, two hundred sixteen TB patients who visited DOTS clinics of two health facilities in Afar Region were included consecutively. Time from onset of symptoms till first consultation of formal health providers (patients’ delay) and time from first consultation till initiation of treatment (health system’s delay) were analyzed.

**Results:**

The median patients’ and health system’s delay were 20 and 33.5 days, respectively. The median total delay was 70.5 days with a median treatment delay of 1 day. On multivariate logistic regression, self-treatment (aOR. 3.99, CI 1.50-10.59) and first visit to non-formal health providers (aOR. 6.18, CI 1.84-20.76) were observed to be independent predictors of patients’ delay. On the other hand, having extra-pulmonary TB (aOR. 2.08, CI 1.08- 4.04), and a first visit to health posts/clinics (aOR. 19.70, CI 6.18-62.79), health centres (aOR. 4.83, CI 2.23-10.43) and private health facilities (aOR. 2.49, CI 1.07-5.84) were found to be independent predictors of health system’s delay.

**Conclusions:**

There is a long delay in the diagnosis and initiation of treatment and this was mainly attributable to the health system. Health system strengthening towards improved diagnosis of TB could reduce the long health system’s delay in the management of TB in the study area.

## Background

According to World Health Organization (WHO), one-third of the world’s population is estimated to be infected with *Mycobacterium tuberculosis*. In 2009, there were 8.8 million new cases with 1.45 million deaths globally; however, the vast majority of Tuberculosis (TB) cases and deaths are from developing countries where resources are limited [1].

Ethiopia carries the highest burden of TB in the Horn of Africa [2] and it stands 8^th^ among the 22 high-burden countries in the world. The national TB notification rate (new and relapse) was 151 per 100,000 population [3]. In response to the problem, Directly Observed Treatment Short Course (DOTS) has been introduced in the country in 1994 and progressively expanded with decentralization of treatment centres. However, with only 60 % of the population accessing health services within 10 km walk [4], management of TB under the DOTS strategy remain a challenge.

Management of TB patients involves early (& accurate) diagnosis, and appropriate treatment to reduce transmission, morbidity, mortality and development of drug resistance. Currently, detection of TB requires that patients are aware of their symptoms and have access to health facilities. Once they come in contact with a health facility, the diagnosis of TB depends on clinical suspicion and subsequent laboratory confirmation which in turn depends on the type of diagnostics available and the skills of the laboratory personnel. In this complex continuum, anything could go wrong and patients may remain undetected leading to delayed initiation of treatment with high morbidity and mortality as well as continued transmission.

Delay in diagnosis and treatment of TB patients could be due to patients’ delay in seeking health care, health care providers’ delay in making prompt and accurate diagnosis with subsequent initiation of treatment or both. Diagnostic delay has been found to be a major obstacle in the control of TB especially in low income countries [5]. A number of factors have been reported to be associated with delay. Some of these factors are negative sputum smear; extrapulmonary TB (EPTB); rural residence; initial visitation of a government low-level health care facility, private practitioner, or traditional healer; and low awareness about TB [5].

In Ethiopia, studies have reported a long delay between the onset of symptoms and initiation of treatment mainly attributable to patient’s delay [6,7]; however, to our knowledge, there is only one report from a pastoralist area with low health service coverage. We, therefore, assessed delay in the diagnosis and treatment of TB patients in two health facilities of Afar Region which is a predominantly pastoralist area.

## Methods

### Study area

Afar Region is one of the 9 regions with estimated area of 100,000 square km [8]. The Region with its 5 zones is home to about 1.4 million people 87 % of which living in rural areas [9]. The Region is arid and semiarid mainly inhabited by pastoralists [10]. The health service coverage in the Region is low with only 40 % of the population accessing health facilities in 10 kilometers walk [10]. The population in Afar Region is affected with a variety of infectious diseases. TB is a major health problem with a notification rate of 103 per 100,000 populations [11].

### Study design and study population

Institution-based cross-sectional study was conducted between September 2009 and March 2010 among TB patients coming to TB clinics of two health facilities in Afar Region. Because of poor infrastructure and security threats in the Region, we selected Dubti hospital and Asayta health centre, both located in Zone one where nearly a third of the Region’s population lives [9]. Both health facilities provide diagnostic and treatment services for various illness including TB. Dubti Hospital is a general hospital with better qualified staff including medical doctors whereas Asayta health centre is mainly staffed with nurses and health officers.

Patients (18 years or older) diagnosed with TB of all forms according to the national TB guideline [12] and came to TB clinics of the two health facilities were included consecutively and interviewed with a semi-structured questionnaire just before commencing treatment. Patients younger than 18 years, those started on treatment prior to interview and those who decline to respond were excluded.

A sample size of 216 was estimated by taking a proportion of 90 % diagnostic delay for more than one month from a study in a similar setting [6], a 95 % confidence interval and a margin error of 4 %

### Operational definitions of variables

Smear positive pulmonary TB (PTB) patients: patients with two or more sputum smears positive for AFB (acid fast bacilli) or one sputum positive for AFB and radiological abnormalities consistent with active TB.

Smear negative PTB patients: Patients with three smears negative for AFB and radiological abnormality consistent with active TB or failure to respond to antibiotic trials.

EPTB patients: TB in organs other than the lungs proven by histo-pathology or TB based on strong clinical evidence consistent with active EPTB and the decision by a physician to treat with a full course of anti-TB therapy.

Patients’ delay: The time interval between date of onset of TB symptoms and first presentation to a professional health provider.

Health system’s delay: The time interval between date of first presentation of patients to a professional health provider and initiation of treatment.

Diagnostic delay: The time interval between the onset of TB symptoms and diagnosis of TB.

Treatment delay: The time interval between date of diagnosis and initiation of treatment.

Total delay: The time interval between date of onset of TB symptoms and initiation of treatment.

Distance: The distance between place of residence and the nearest health facility at the time of illness.

Non-formal health providers: These include traditional health providers, local injectors and drug retail outlets.

Formal health providers: Professional health providers working in modern health facilities i.e. hospitals, health centres, clinics owned by the government or the private sector.

Pastoralist: People whose source of livelihood is livestock with which they move seasonally in search of pasture and water.

Self-treatment: Any kind of self-prescribed treatment taken by patients for their illness.

### Data collection

Pre-tested, semi-structured questionnaire was used to collect data on socio-demographic characteristics; distance of residence at the time of illness from the nearest health facility; knowledge on TB, its cause, its seriousness and its treatment (able to mention bacteria/germ as a cause of TB, able to classify TB as a transmissible disease, able to tell that untreated TB could lead to death, able to mention that TB is a treatable disease, able to mention the approximate duration of treatment, knows that the drugs are available at health facilities for free); time interval between onset of symptoms and first visit to a health facility; time interval between first visit of a health facility and diagnosis; and time interval between diagnosis and treatment were collected. One nurse at each TB clinic conducted the interview after training.

The date of onset for the main symptoms was taken as the date of onset for the illness. For PTB patients, cough was taken as the main symptom whereas for EPTB patients, either localizing symptoms like swelling for TB lymphadenitis, chest pain for TB pleurisy or constitutional symptoms (fever, night sweats, weight loss, loss of appetite) were taken as the onset of the illness whichever came first.

### Data analysis

Data was analyzed using SPSS for Windows version 16. Since data were skewed, non- parametric tests (Mann–Whitney/Kruskal Wallis) were used in evaluating group differences. In some studies, experts agreed 30 days as acceptable cut-off for delay [13,14] whereas others used median value of the observed data as a cut-off [6,15,16] and we adopted the later to dichotomize data into delayed and not delayed. Responses to questions to assess TB knowledge were analyzed by calculating their mean and interquartile scores. Using the mean score as a cut- off, the responses were categorized into good (equal or above the mean) and poor knowledge (below the mean) and were cross-tabulated with the main outcome variables for possible associations.

Independent variables that are either significantly associated with the dependent variables on bivariate analysis or are known to be associated with the dependent variables from previous studies were selected and multivariate logistic regression was done to identify independent predictors of dependent variables. The association of predictor variables with the dependent variables was assessed by using 95 % confidence interval (CI) and adjusted odds ratio (aOR). A p-value of < 0.05 was considered statistically significant.

### Ethics statement

The study has been ethically approved by the Norwegian Ethics Committee (Regionale komiteer for medisinsk og helsefaglig forskningsetikk (2009/284a)) and Ethics Committee of Aklilu Lemma Institute of Pathobiology, Addis Ababa University (01/2000 Ethiopian Calendar). Patients fulfilling the inclusion criteria were invited to participate in the study and written consent was obtained.

## Results

### Socio-demographic characteristics of patients

A total of 216 TB patients were included in this study. The response rate was 78 % and the non- responders were not significantly different from responders with regard to basic socio- demographics. The mean age was 32.7 + 12.3 years with a male to female ratio of 1.67 to 1.The vast majority (83.8 %) of study participants were under 45 years of age (Table 1).

**Table 1:** Association of socio-demographic and health related factors with patients’ delay, Afar Region, Ethiopia

**Characteristics**	**Number**	**% Delayed ^Ψ^**	**aOR (95 % CI)**
Age			
18-24	54	42.6	1.00
25-44	127	42.5	1.20 (0.52-2.78)
>44	35	65.7	3.00 (0.99-9.12)
Sex			
Male	135	48.1	1.00
Female	81	43.2	1.01 (0.48-2.11)
Marital status			
Single	68	42.6	1.00
Married	119	49.6	0.83 (0.37-1.86)
Widowed	14	35.7	0.48 (0.10-2.21)
Divorced	15	46.7	1.22 (0.33-4.48)
Occupation			
Non-pastoralist	125	36.0	1.00
Pastoralist	91	60.4	1.91 (0.90-4.05)
Education			
None	140	53.6	1.00
Primary	53	37.7	0.82 (0.37-1.83)
Post-primary	23	21.7	0.52 (0.15-1.79)
Biomedical knowledge			
Poor	34	55.9	1.00
Good	182	44.5	0.78 (0.30-1.86)
Distance to facility			
≤ 10 km	163	41.7	1.00
>10 km	53	60.4	1.33 (0.62-2.86)
Form of TB			
PTB	137	43.8	1.00
EPTB	79	50.6	1.52 (0.80-2.87)
Self treatment			
No	186	41.4	1.00
Yes	30	76.7	3.99 (1.50-10.59)*
First health action			
Formal health provider	191	41.4	1.00
Non-formal provider	25	84.0	6.18 (1.84-20.76)*

Ψ Delayed more than 20 days.

*Significantly associated.

The majority (75 %) of study participants reported to live within 10 km radius from the nearest health facility with a median of 3 km. The median distance was 3 km (13.3 km for pastoralists and 2.5 km for non-pastoralists). Regarding the biomedical knowledge of participants on the causes, treatment and outcome of TB, 84.3 % of participants had good knowledge.

### Forms of TB and symptoms reported

Regarding the forms of TB, 137 (63.4 %) had PTB and the rest were diagnosed with EPTB. Among PTB patients, the majority (61.3 %) of them were diagnosed as smear negative PTB.

Patients with PTB reported persistent cough (100 %), fever (93.4 %), weight loss (92 %), loss of appetite (89.1 %), night sweating (84.7 %), and haemoptysis (26.3 %). On the other hand, patients with EPTB reported fever (89.9 %), swelling (mainly neck and axillary areas) (73.4 %), loss of appetite (77.2 %), weight loss (77.2 %), night sweating (64.6 %), chest pain (25.3 %), and cough (17.7 %).

### Health seeking action of study participants

First health seeking action of study participants and associated factors were further investigated after categorizing health providers as formal and non-formal. With regard to first visit, the majority (88.4 %) of study participants consulted formal health providers and the rest (11.6 %) sought help from non-formal health providers. There was no significant difference with regard to participants’ first health action by age, sex, occupation, education, distance and form of TB. A significantly higher proportion of rural dwellers (19.2 %) compared to urban dwellers (7.7 %) initially visited non-formal health providers (p = 0.01). Similarly, a significantly higher proportion of those with self-treatment (30 %) compared to those without self-treatment (8.6 %) visited non- formal providers (p < 0.01).

### Comparison of pastoralists and non-pastoralists

Differences between pastoralists and non-pastoralists in relation to socio-demographic characteristics, forms of TB, distance to the nearest health facility and first health seeking action were compared. The two groups did not differ significantly with regard to age, form of TB and first health seeking action. However, the proportion of males, those with no education, and those living more than 10 km from health facilities were significantly higher among pastoralists compared to their respective proportions among non-pastoralists (p < 0.001).

### Patients’ delay

The median patients’ delay was 20 (IQR 8–60) days; it is 15 days for patients with PTB and 21 days for patients with EPTB. About 76 % of participants sought help within one month. On bivariate analysis, age was significantly associated with patients’ delay (Kruskal Wallis, p = 0.042). The median patients’ delay for those patients who initially visited formal health providers (15 days) was significantly shorter compared to those who first consulted non-formal health providers (60 days) (Mann Whitney, P < 0.001). Similarly, patients with self-treatment reported a significantly longer delay (52.5 days) compared to those without self-treatment (15 days) (Mann Whitney, p < 0.001). Moreover, the median patients’ delay for pastoralists was significantly longer compared to that of non-pastoralists (30 days versus 15 days) (Mann Whitney, p < 0.001).

With regard to knowledge about TB, the median delay for those with good knowledge was 15 days compared to a 30 days median delay for those with poor knowledge (Mann Whitney, p = 0.06). Those who reported to live more than 10 km away from the nearest health facility experienced a significantly longer delay (median of 30 days) compared to those who reported to live within 10 km (median of 15 days) (Mann Whitney, p = 0.018). On multivariate logistic regression, self-treatment (aOR. 3.99, CI 1.50-10.59) and first visit to non-formal health providers (aOR. 6.18, CI 1.84-20.76) remained as predictors of patients’ delay (Table 1).

### Health system’s delay

The median health system’s delay was 33.5 (IQR 17–87) days. About 70 % of patients were diagnosed only after the second or the third visit.

On bivariate analysis, form of TB (Mann Whitney, p = 0.016) and the type of health facility initially visited (Kruskal Wallis, p < 0.001) were found to be significantly associated with health system’s delay. This association remained significant on multivariate logistic regression (Table 2); those with EPTB had a significantly longer health system’s delay compared to those with PTB (aOR. 2.08, CI 1.08- 4.04). Similarly, taking government hospital as a reference, first visit to government clinics/health post (aOR. 19.70, CI 6.18-62.79), health centres (aOR. 4.83, CI 2.23-10.43), and private health facilities (aOR. 2.49, CI 1.07-5.84) were independent predictors of longer health system’s delay.

**Table 2:** Association of socio-demographic and health-related factors with health system’s and total delay in Afar Region, Ethiopia

**Characteristics**	**Number**	**Health system’s delay**		**Total delay**	
		**% Delayed^†^**	**aOR (95 % CI)**	**% Delayed^‡^**	**aOR (95 % CI)**
Age					
18-24	54	50.0	1.00	48.1	1.00
25-44	127	54.3	1.24 (0.58-2.62)	52.8	1.16 (0.58-2.35)
>44	35	34.3	0.64 (0.22-1.83)	42.9	0.87 (0.34-2.26)
Sex					
Male	135	48.1	1.00	49.6	1.00
Female	81	53.1	1.33 (0.67-2.62)	50.6	1.04 (0.56-1.95)
Education					
None	140	47.9	1.00	52.9	1.00
Primary	53	54.7	1.68 (0.74-3.84)	49.1	1.14 (0.53-2.43)
Post-primary	23	52.2	1.67 (0.54-5.18)	34.8	0.70 (0.24-2.04)
Occupation					
Non-pastoralists	125	48.8	1.00	45.6	1.00
Pastoralists	91	51.6	1.68 (0.77-3.68)	56.0	1.40 (0.69-2.86)
Distance					
≤ 10 km	163	50.3	1.00	47.9	1.00
>10 km	53	49.1	0.78 (0.35-1.74)	56.6	1.11 (0.53-2.31)
Form of TB					
PTB	137	43.8	1.00	41.6	1.00
EPTB	79	60.8	2.08 (1.08-4.04)*	64.6	2.56 (1.39-4.73)^*^
Self-treatment					
No	186	52.7	1.00	48.9	1.00
Yes	30	33.3	0.51 (0.20-1.32)	56.7	1.42 (0.61-3.29)
Health provider					
Hospital	68	23.5	1.00	45.6	1.00
Health centre	69	63.8	4.83 (2.33-10.43)^*^	55.1	1.28 (0.63-2.61)
Clinic/health post	32	84.4	19.70 (6.18-62.79)^*^	65.6	2.55 (1.01-6.03)^*^
Private facility	47	44.7	2.49 (1.07-5.84)^*^	38.3	0.82 (0.37-1.82)

^†^Delayed more than 33.5 days.

^‡^Delayed more than 70.5 days.

*Significantly associated.

### Treatment delay

In this study, we found a median treatment delay of 1 day (IQR 1–4 days). The median treatment delay was significantly longer among those diagnosed at private health facilities (4 days) compared to those diagnosed at government health facilities (1 day) (Mann–Whitney test, p < 0.001). Ninety-eight percent of patients diagnosed at government health facilities were started on treatment within 5 days; however, almost 22 % of patients diagnosed at private health facilities were started on treatment after 5 days.

### Total delay

The median total delay was 70.5 (IQR 37–126.75) days. Only 5.1 % of patients started treatment within one month following onset of symptoms. Health system’s delay contributed a greater proportion of the total delay. Health system’s delay was longer than patients’ delay for 149 (69.0 %) patients. Interestingly, patients who consulted the health system earlier experienced a longer health system’s delay. Patients who visited a health facility within 20 days following the onset of their illness had a significantly longer median health system’s delay (49 days) compared to those who consulted a health facility after 20 days (29 days) (Mann–Whitney, p < 0.001). On univariate analysis, total delay was not significantly associated with age, sex, marital status and education. Although it did fail to reach statistical significance (p = 0.09), those who lived more than 10 km from health facilities had a longer median total delay (93 days) compared to those who lived within 10 km radius (67 days). On the other hand, the first formal health provider visited was found to be significantly associated with total delay (Kruskal-Wallis, p = 0.03). Similarly, pastoralists (Mann–Whitney, p = 0.01) and patients with EPTB (Mann- Whitney, p = 0.002) had a significantly longer total delay.

On multivariate logistic regression analysis, having EPTB (aOR. 2.56, CI 1.39-4.73) and first visit to government health posts/clinics (aOR. 2.55, CI 1.01-6.03) remained independent predictors of longer total delay (Table 2).

## Discussion

Early detection and effective treatment are key strategies to control TB. Early detection would be possible if TB patients report early enough and the health system detects TB patients within a reasonable time. We assessed delay and associated factors in the diagnosis and treatment of TB in Afar Region, Ethiopia.

### Patients’ delay

In the current study, the median patients’ delay was 20 days. At least 50 % of PTB patients were able to consult health facilities within 2 weeks which is generally the timeline advised for a patient with cough to visit a health facility. The short median patient delay in the current study is in sharp contrast to a report from another pastoralist area in Ethiopia (6).

Compared to previous studies in Ethiopia [7,17,18], the median patients’ delay for all forms of TB in the current study is short. Moreover, a lower proportion of our participants sought help from non-formal health care providers in contrary to previous reports from Ethiopia [6,15,18]. However, unlike our study, two of these studies [6,18] have reported a poor knowledge among study participants about TB. A recent study in Afar Region on knowledge and perception of pastoralist communities about TB reported a high degree of awareness [19] which is consistent with our finding. The high degree of awareness among our study participants about TB might be a possible reason for a shorter patients’ delay as well as their preference to formal health care providers. Information is highly valued among the Afar people and they heavily depend on a traditional face-to-face communication which is called *dagu *[20]. It might be possible that information related to TB might have been channelled through *dagu* resulting in high awareness about the disease.

In the current study, first visit to a non-formal health provider was found to be an independent predictor of patients' delay, consistent with reports from Ethiopia [15,18], Tanzania [13] and Thailand [21]. In our study, those patients who consulted non-formal health providers initially have actually did that early enough (median of 10 days); this is a good opportunity to decrease patient-related delay if attention is given to non-formal health providers’ awareness on TB so they could refer TB suspects to formal health providers early. Similarly, we found self-treatment as an independent predictor of patient delay which is consistent with a previous report from Ethiopia [15]. Those patients who treated themselves might consult formal health care providers only after the disease gets worsened. In agreement with this, a qualitative study in Kenya reported that treatment steps for TB symptoms are generally sequential and the usual initial response of patients to an illness is self-treatment [22].

### Health system’s delay

The median health system’s delay in our study was longer compared to previous reports from Ethiopia [6,7,15]. Our participants presented themselves early in the course of their illness which might pose a diagnostic challenge to the health system staffed with low level health workers.

In the present study, EPTB was found to be an independent predictor for health system’s delay which is consistent with previous reports from Ethiopia [6], Norway [23] and London [24]. This is possibly due to that fact that diagnosis of EPTB especially at low level health facilities is challenging. Patients might be treated with several doses of antibiotics before diagnosis and commencement of anti-TB medication. Although patients with EPTB do not pose a threat to the community in terms of transmission, they could suffer long term disability, high morbidity and mortality as a result of late diagnosis.

Those patients who first visited government health posts/clinics and health centres had a significantly longer delay compared to those who first visited a government hospital and this is consistent with previous reports from Ethiopia [15] and Botswana [25]. According to a review, the main problem with regard to delay seems to be related to “a vicious cycle of repeated visits of the same health care level, resulting in non-specific antibiotic treatment and failure to access specialized TB services” [5]. In Ethiopia, government health posts/clinics do not have TB diagnostic laboratories; besides, health facilities in Afar Region have a poor staffing quality with weak supervision [26]. In such situations, it is likely that TB patients be treated with several doses of antibiotics before TB is suspected leading to delayed diagnosis.

First visit to private health facilities was found to be an independent predictor of longer delay and this is in agreement with previous reports [15,21,27]. Private health facilities were not part of the DOTS programme until very recently and it is likely that health workers could have low awareness on the management of TB patients.

The median treatment delay in our study was 1 day, consistent with a report from Ghana [28]. In this study, the treatment delay (median of 1) for patients diagnosed at government health facilities is acceptable. However, the median treatment delay for those patients who were diagnosed at private health facilities was 4 days, significantly longer than the median delay for those diagnosed at government health facilities. This might be related to the fact that private health facilities, mainly found in Dessie, are far from patients’ residence and following diagnosis, patients are referred back to the nearest health facility to their residence. Improved diagnostic services at lower government health facilities coupled with increased awareness of the public on the availability of such services closer to their residence could avoid unnecessary delay after diagnosis is made.

### Total delay

The median total delay in our study was found to be 70.5 days and this is in good agreement with previous reports from Ethiopia [6,7,15] as well as Nigeria [29] and Thailand [21]. In the current study, health system’s delay was a major contributor to the total delay, consistent with a previous report from Ethiopia [15]. Interestingly, those who visited the health system earlier experienced a longer health system’s delay and this is consistent with the finding from Botswana [25]. Contrary to the findings of this study, two studies from Ethiopia [6,7] reported a long median patients’ delay (60 days) and a short health system’s delay (6 days). The index of suspicion could be low in those patients presented early and health workers may entertain other alternative diagnosis. Further research exploring factors for delayed diagnosis of those patients who visited health facilities earlier is important.

The current study has limitations. First, we have included patients who came to the DOTS clinics of two health facilities. However, TB patients who visited other facilities as well as those who didn’t report to a health facility during the study period might have experienced longer delay. This influences the representativeness with possible underestimation of the length of delay we reported and therefore it is difficult to generalize the result to all TB patients in the Region. Secondly, the duration of delay was based on self-report implying recall bias. We have used religious dates as a reference to minimize recall bias among participants.

## Conclusions

The majority of TB patients in the current study reported to the health system in a relatively short time. However, there is a long delay in the diagnosis and initiation of treatment mainly because of the poor response of the health system. Training, supervision and continued support of health workers together with availing improved TB diagnostic services is needed to reduce delay in the diagnosis of TB. Moreover, decentralization of the DOTS programme to the lower health facilities might help in reducing delay in diagnosis as well as initiation of treatment of TB.

## Abbreviations

aOR, Adjusted Odds Ratio; CI, Confidence Interval; DOTS, Directly Observed Treatment Short course; EPTB, Extra-Pulmonary TB; IQR, Interquartile Range; PTB, Pulmonary TB; TB, Tuberculosis; WHO, World Health Organization.

## Competing interests

The authors declared that they have no competing interests.

## Authors’ contributions

MB: Involved in the design, data collection, statistical analysis and interpretation of data; drafted the manuscript, FA: Involved in the design, data analysis, interpretation and critical revision of the manuscript GB: Involved in the design, interpretation and critical revision of the manuscript, GA: Involved in data collection and critical revision of the manuscript All authors have read and approved the manuscript for submission.

## Pre-publication history

The pre-publication history for this paper can be accessed here:

http://www.biomedcentral.com/1471-2458/12/369/prepub
